# Bacteria under antibiotic attack: Different strategies for evolutionary adaptation

**DOI:** 10.1371/journal.ppat.1008431

**Published:** 2020-05-07

**Authors:** Etthel M. Windels, Bram Van den Bergh, Jan Michiels

**Affiliations:** 1 VIB Center for Microbiology, Flanders Institute for Biotechnology, Leuven, Belgium; 2 Centre of Microbial and Plant Genetics, KU Leuven, Leuven, Belgium; University of Massachusetts, Worcester, UNITED STATES

## Abstract

Bacteria are well known for their extremely high adaptability in stressful environments. The clinical relevance of this property is clearly illustrated by the ever-decreasing efficacy of antibiotic therapies. Frequent exposures to antibiotics favor bacterial strains that have acquired mechanisms to overcome drug inhibition and lethality. Many strains, including life-threatening pathogens, exhibit increased antibiotic resistance or tolerance, which considerably complicates clinical practice. Alarmingly, recent studies show that in addition to resistance, tolerance levels of bacterial populations are extremely flexible in an evolutionary context. Here, we summarize laboratory studies providing insight in the evolution of resistance and tolerance and shed light on how the treatment conditions could affect the direction of bacterial evolution under antibiotic stress.

## Strategies to overcome antibiotic treatment

Bacterial populations can adopt different strategies to become refractory to an antibiotic treatment that would otherwise be lethal. The emergence and dissemination of these survival strategies can be the result of either vertical transmission of de novo mutations or horizontal transfer of mobile genetic elements. As horizontal gene transfer (HGT) has proved challenging to track during laboratory evolution, the environmental parameters that affect it remain poorly understood. Despite the importance of HGT, this Review therefore mainly focuses on evolution by de novo mutations, which can easily be monitored in the lab.

A well-documented antibiotic survival strategy is resistance, which is usually conferred by genetic changes that allow bacteria to grow at elevated drug concentrations. Resistance is routinely quantified by the minimum inhibitory concentration (MIC), defined as the lowest antibiotic concentration that is required to prevent bacterial growth [1]. However, even populations with a low MIC may display considerable survival when facing antibiotic attack. This is, in many cases, due to antibiotic tolerance, which allows bacteria to survive but not proliferate during a high-dose antibiotic treatment [2]. In contrast to resistant mutants, tolerant cells only differ phenotypically from susceptible cells and can constitute the entire population or be present as a subpopulation of cells. Tolerance in only a fraction of the population is referred to as persistence [3,4]. Throughout the remainder of the text, we will consider persistence as a specific case of tolerance, and we will not make the distinction, since both are similar regarding the phenotype and the evolutionary and clinical consequences.

While resistance is specifically expressed in terms of antibiotic concentrations, tolerance only weakly depends on the applied concentration. Tolerant cells are killed much more slowly than susceptible cells in a broad range of antibiotic concentrations, implying that tolerance levels can be quantified based on the minimum duration for killing (MDK) of a population at high concentrations [5].

Bacteria are notorious for their high adaptive potential when facing different types of stress, including antibiotic therapy. Genetic changes can cause increased levels of resistance or tolerance, either directly or by affecting the expression of other resistance or tolerance genes [6], thereby allowing bacterial populations to better cope with the antibiotics to which they are exposed. Since recently, this adaptability is being extensively explored using experimental evolution.

## Evolution to high resistance

As a result of the imminent threats posed by antibiotic-resistant pathogens, much research has been devoted to the emergence and evolution of resistance in the face of antibiotic treatment. These studies often benefit from laboratory evolution experiments that mimic bacterial adaptation during an antibiotic therapy, enabling real-time observation of evolutionary changes and detailed reconstruction of mutational trajectories. Resistance often evolves by exposing bacteria to a fixed dose of an antibiotic. However, as the selection pressure declines once a single resistance mutation is acquired, this setup mostly results in low-level resistance and confers only limited insight in the evolutionary dynamics of higher-level resistance. Maintaining the selection pressure by gradually increasing the antibiotic concentration in space [7,8] or time [9–12] facilitates the accumulation of multiple resistance mutations and often results in highly resistant mutants, thereby mimicking evolutionary dynamics in clinical environments characterized by spatial and/or temporal heterogeneity. Theory predicts that selection for resistance is strongest when the antibiotic concentration is above the MIC, as these conditions completely suppress growth of susceptible cells and thus maximize the growth advantage of resistant mutants [13]. However, since single-step mutants often have only slightly elevated MICs, they are unlikely to arise when the antibiotic concentration exceeds a threshold called the mutant prevention concentration (MPC) [13]. Sub-MIC drug concentrations on the other hand, still exert deleterious effects on susceptible cells, thereby also resulting in a strong selective enrichment of highly resistant, clinically relevant mutants [11,14–16].

Antibiotic resistance is often associated with a fitness cost due to the maintenance of resistance mechanisms and/or mutations in genes that play an essential role in bacterial metabolism. This fitness defect is usually reflected as a reduced growth rate in antibiotic-free conditions [17]. Notably, a recent study suggests that resistant mutants arising under sublethal antibiotic stress exhibit smaller fitness defects than those emerging at high doses, due to stronger competition with susceptible cells [18].

Experimental evolution is a highly effective tool to investigate resistance evolution by de novo mutations. In addition to revealing evolutionary dynamics of resistance to a single drug, it also facilitates the investigation of different drug combinations and their effect on resistance development, which can provide novel options for treatment [19–21]. Nevertheless, a significant proportion of bacterial pathogens acquires (multidrug) resistance through HGT, which is challenging to monitor during experimental evolution. Some efforts have already been made in this field [22], yet novel approaches that shed light on the emergence and spread of resistance transferred on mobile genetic elements would yield valuable insights in the dynamics of infection.

In addition to the numerous in vitro evolution experiments, several studies have focused on antibiotic resistance evolution within a host. Whereas an appropriate in vivo model for resistance evolution is currently still lacking, large collections of longitudinal clinical isolates have been sequenced and phenotypes have been assessed, revealing dynamics of within-host resistance evolution as well as patient-to-patient transmission of resistance genes [23–25]. It has become clear that in vitro rates of resistance emergence often do not adequately predict resistance development in clinical settings, due to the involvement of HGT, distinct environmental conditions and bacterial population sizes, in vivo fitness costs, multispecies interactions, and other factors that are overlooked in laboratory studies [26]. Unraveling the evolutionary dynamics of clinical resistance requires more extensive longitudinal studies and could be further supported by the systematic collection of antibiotic sensitivity data of within-host pathogens as well as commensal species [27]. Combined, evolutionary insights obtained from laboratory evolution experiments and clinical data are indispensable to diagnose and prevent the development of antibiotic resistance during infections.

## Evolution to high tolerance

In contrast to genetic resistance, the evolvability of antibiotic tolerance has only recently been explored. Theoretical models predict that a periodic high-dose treatment, a schedule that is often adopted in clinical practice, should initially favor the emergence of high tolerance [28,29]. These predictions have been complemented by data from multiple independent evolution experiments. When repeatedly diluting an overnight culture of *Escherichia coli* in fresh medium containing a high ampicillin dose, mutants are selected that exhibit population-wide tolerance conferred by an increased lag time that is protective against the antibiotic [5]. Furthermore, when populations of *E*. *coli* [30–32], *Staphylococcus aureus* [33], or ESKAPE pathogens (*Enterococcus faecium*, *S*. *aureus*, *Klebsiella pneumoniae*, *Acinetobacter baumannii*, *Pseudomonas aeruginosa*, and *Enterobacter* spp.) [34] are treated daily with a high concentration of antibiotics, the fraction of tolerant cells within the population rapidly increases. Worryingly, populations evolved under these conditions harbor approximately 1000-fold more tolerant cells after only a few treatment cycles. Although similar evolution experiments have not yet been performed in vivo, longitudinal isolates of cystic fibrosis patients with chronic *P*. *aeruginosa* infections display increasing tolerance levels over time [35]. These observations suggest that evolution of antibiotic tolerance also occurs within a patient and can therefore no longer be overlooked in clinical practice.

## Evolution of resistance and tolerance: Alternative routes to antibiotic survival?

**Fig 1:**
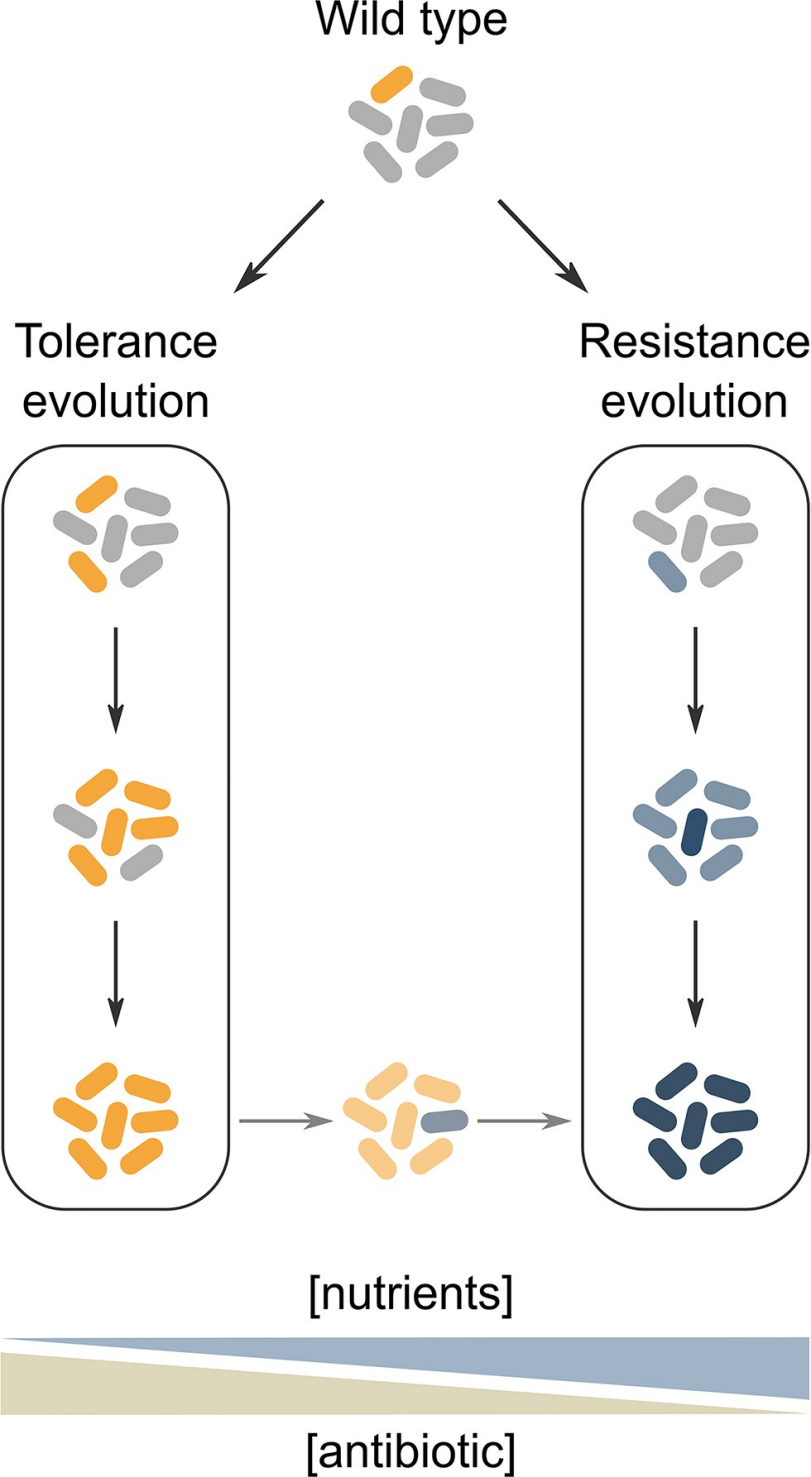
Conceptual model of tolerance and resistance evolution in different antibiotic treatment conditions. Tolerance is presumably favored when high antibiotic concentrations are applied in growth-restricting conditions (left). On the other hand, resistance is mainly favored when the nutrient concentration is sufficiently high to allow growth, while the antibiotic concentration should not be too high to be overcome by resistance mutations (right). In conditions that select for resistance, a tolerant strain evolves resistance faster than a wild-type strain. It is currently unclear which survival strategy is favored in conditions characterized by other combinations of antibiotic and nutrient concentrations.

Resistance and tolerance both provide a considerable selective advantage to populations that are frequently exposed to antibiotics. Yet depending on the antibiotic treatment schedule and the environmental conditions, either resistance or tolerance might be the most beneficial survival strategy. How these conditions affect the direction of evolutionary adaptation is a poorly studied subject, but some hypotheses can be inferred from the properties of each survival strategy (Fig 1).

Resistance mutations allow cells to grow during antibiotic treatment, while they are often associated with a fitness cost in antibiotic-free conditions. This implies that resistance is mainly beneficial for populations that are continuously exposed to antibiotics. At the same time, other environmental parameters (e.g., nutrient concentrations), should be favorable for growth in order to maximize the selective advantage of resistant mutants over susceptible cells, which are either growth-inhibited or killed by the antibiotic. As resistance is acquired by the stepwise accumulation of mostly low-effect mutations, initial selection should occur in sufficiently low antibiotic concentrations in order to obtain evolution at all.

Tolerance, on the other hand, allows cells to survive high doses of antibiotics, without enabling growth during treatment. Increased tolerance is therefore expected to be the favorable evolutionary route when antibiotic treatment occurs in an environment that does not promote growth (for example, in nutrient-limiting conditions). Moreover, while tolerance mutations might also be associated with growth defects in an antibiotic-free environment [30,36], the antibiotic-tolerant phenotype is in many cases mainly expressed in growth-inhibiting conditions [37]. Despite these theoretical arguments, the effect of nutrient concentrations on the evolution of tolerance remains to be determined experimentally. Antibiotic doses that are difficult to overcome by a single resistance mutation are also likely to select for tolerance, which only weakly depends on the concentration. Tolerance evolution requires such high-dose treatments to be intermitted with periods of growth in order to allow bacterial division and thus selection. Whether evolved populations exhibit tolerance as a whole or only in a subpopulation of cells presumably depends on the frequency of antibiotic treatment. The percentage of tolerant cells in evolved populations has indeed been shown to negatively correlate with the time interval between two treatments [30]. Similarly, other bacterial parameters, such as the duration of population-wide tolerance [5], can be fine-tuned to match the duration of antibiotic treatment.

Resistance and tolerance are often considered as alternative, mutually exclusive survival strategies, each with benefits and costs associated to specific environmental conditions. Nevertheless, various recent studies have shown that acquiring a tolerance mutation does not constrain but even accelerates subsequent resistance evolution in conditions that are favorable for resistance [38–41]. Similar observations have recently been made in a patient in which tolerance to a combination treatment fueled resistance development [42]. Furthermore, a positive epistatic interaction has been demonstrated between resistance and tolerance mutations for survival under antibiotic treatment [43]. Although observations in *Pseudomonas* isolates suggest that natural environments can promote the selection of both resistance and tolerance [44], coevolution of both traits has not yet been observed in real time.

The above-mentioned concepts might provide a basis to predict bacterial adaptation to antibiotic stress within a patient, as a function of the local concentrations of nutrients and antibiotics. Problematically however, in vivo environments are extremely complex, heterogeneous, and not well characterized, which considerably complicates the extrapolation of in vitro observations to within-host conditions [45,46]. Indeed, local concentrations of nutrients and antibiotics vary strongly among and even within tissues. Moreover, additional environmental parameters, such as physicochemical properties, compartmentalization, and the presence of immune cells, presumably also play a role in in vivo growth and evolution of bacteria [45]. Several tools have recently been developed to characterize the local environment that is sensed by bacteria during an infection [47–50], which can be mimicked in laboratory evolution experiments. Furthermore, most laboratory studies focus on single-drug treatments, while patients in the clinic are often treated with combinations of antibiotics. A recent study showed that combination treatments strongly affect the evolution of tolerance and resistance, thereby highlighting the need to bridge this gap [42]. Overall, future research should be directed to in vivo evolution in order to reconstruct real-world evolutionary trajectories and identify environmental parameters that affect the direction of evolution.
